# Molecular network, pathway, and functional analysis of time-dependent gene changes associated with pancreatic cancer susceptibility to oncolytic vaccinia virotherapy

**DOI:** 10.1038/mto.2016.8

**Published:** 2016-03-16

**Authors:** Dana Haddad, Nicholas Socci, Chun-Hao Chen, Nanhai G Chen, Qian Zhang, Susanne G Carpenter, Arjun Mittra, Aladar A Szalay, Yuman Fong

**Affiliations:** 1 Department of Surgery, Memorial Sloan-Kettering Cancer Center, New York, New York, USA;; 2 Department of Biochemistry, University of Wuerzburg, Wuerzburg, Bavaria, Germany;; 3 Bioinformatics Core Facility, Memorial Sloan-Kettering Cancer Center, New York, New York, USA;; 4 Genelux Corporation, San Diego Science Center, San Diego, California, USA;; 5 Department of Radiation Oncology, University of California, San Diego, California, USA;; 6 Department of Surgery, City of Hope Medical Center, Los Angeles, California, USA.

## Abstract

Background: Pancreatic cancer is a fatal disease associated with resistance to conventional therapies. This study aimed to determine changes in gene expression patterns associated with infection and susceptibility of pancreatic cancer cells to an oncolyticvaccinia virus, GLV-1h153, carrying the human sodium iodide symporter for deep tissue imaging of virotherapy.

Methods: Replication and susceptibility of pancreatic adenocarcinoma PANC-1 cells to GLV-1h153 was confirmed with replication and cytotoxicity assays. PANC-1 cells were then infected with GLV-1h153 and near-synchronous infection confirmed via flow cytometry of viral-induced green fluorescent protein (GFP) expression. Six and 24 hours after infection, three samples of each time point were harvested, and gene expression patterns assessed using HG-U133A cDNA microarray chips as compared to uninfected control. Differentially expressed genes were identified using Bioconductor LIMMA statistical analysis package. A fold change of 2.0 or above was used as a cutoff, with a *P* value of 0.01. The gene list was then analyzed using Ingenuity Pathways Analysis software.

Results: Differential gene analysis revealed a total of 12,412 up- and 11,065 downregulated genes at 6 and 24 hours postinfection with GLV-1h153 as compared to control. At 6 hours postinfection. A total of 139 genes were either up or downregulated >twofold (false discovery rate < 0.05), of which 124 were mapped by Ingenuity Pathway Analysis (IPA). By 24 hours postinfection, a total of 5,698 genes were identified and 5,563 mapped by IPA. Microarray revealed gene expression changes, with gene networks demonstrating downregulation of processes such as cell death, cell cycle, and DNA repair, and upregulation of infection mechanisms (*P* < 0.01). Six hours after infection, gene changes involved pathways such as HMGB-1, interleukin (IL)-2, IL-6, IL-8, janus kinase/signal tranducer and activator of transcription (JAK/STAT), interferon, and ERK 5 signaling (*P* < 0.01). By 24 hours, prominent pathways included P53- and Myc-induced apoptotic processes, pancreatic adenocarcinoma signaling, and phosphoinositide 3-kinase/v-akt murine thymoma vial oncogene homolog 1 (PI3/AKT) pathways.

Conclusions: Our study reveals the ability to assess time-dependent changes in gene expression patterns in pancreatic cancer cells associated with infection and susceptibility to vaccinia viruses. This suggests that molecular assays may be useful to develop safer and more efficacious oncolyticvirotherapies and support the idea that these treatments may target pathways implicated in pancreatic cancer resistance to conventional therapies.

## Background

Oncolytic viral therapies have shown such success in preclinical trials as a novel cancer treatment modality that several phase 1 and 2 trials are already underway.^1^ We have previously reported on the construction and generation of a novel attenuated replication-competent vaccinia virus (VACV), GLV-1h153, a derivative of parental virus GLV-1h68 engineered to carry the human sodium iodide symporter (hNIS) for the imaging of viral replication within tumors via enhanced uptake of several radionuclide probes.^2^ The noninvasive tracking of virus delivery may enable clinicians to correlate efficacy and therapy, monitor potential viral toxicity, and possibly provide a more sensitive and specific diagnostic technique to detect tumor origin and, more importantly, presence of metastases.^3,4^ GLV-1h153 facilitated enhanced dose-dependent radiouptake in cell culture and effective replication and killing of pancreatic cancer cells both in cell culture and in animal models. Furthermore, GLV-1h153 facilitated enhanced uptake in tumors which was readily detected by positron emission tomography.

In this study, we conducted gene expression analysis using cDNAGeneChip microarray Human Genome U133A (Affymetrix, Santa Clara, CA) to determine changes in gene expression patterns over time associated with infection and susceptibility of pancreatic cancer cells to GLV-1h153. Understanding into the molecular mechanisms associated with sensitivity to GLV-1h153 may enable identification of cancers resistant to viral therapy, thus avoiding undesirable side effects associated with the need for higher doses of viral treatment. Furthermore, knowledge of these mechanisms may be useful to develop safer and more efficacious oncolytic virotherapies.

## Results

### GLV-1h153 replication was assessed via flow cytometric detection of GFP

**Figure 1:**
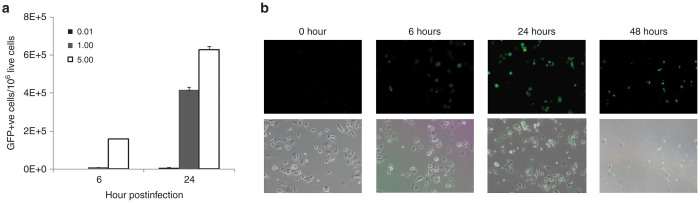
Infection of pancreatic cells and flow cytometry analysis. (**a**) Infection of pancreatic cells to show hNIS protein expression by GLV-1h153, PANC-1 cells were mock infected or infected with GLV-1h153 or parental virus GLV-1h68 at MOIs of 0.1, 1.0, and 5.0 and harvested 24 hours after infection. Results reflect an average of three samples with standard error. (**b**) Viral infection, replication, and cell viability were successfully visualized by assessing GFP expression and was time dependent. Phase overlay pictures shows GFP expression as early as 6 hours post infection with an MOI of 5, with maximal GFP expression after by 24 hours, and cell death and decline of GFP expression by 48 hours. Cells are demonstrated at ×20 magnification.

GFP expression in cells infected with GLV-1h153 was quantified using flow analysis and was shown to be both time and multiplicity of infection (MOI) dependent. Almost 70% of live cells expressed GFP at an MOI of 5.0 at 24 hours postinfection (Figure 1a). Viral infection, replication, and cell viability were successfully visualized by assessing GFP expression and were time dependent. Phase overlay pictures shows GFP expression as early as 6 hours postinfection with an MOI of 5, with maximal GFP expression after by 24 hours, and cell death and decline of GFP expression by day 2 (Figure 1b). Based on flow cytometry and visualization of GFP expression, we harvested cells after infection with an MOI of 5 at 0, 6, and 24 hours postinfection. A near-synchronous infection rate was achieved without cell death and lysis occurring too early for harvest.

### Identification of time-dependent gene-fold changes

**Figure 2:**
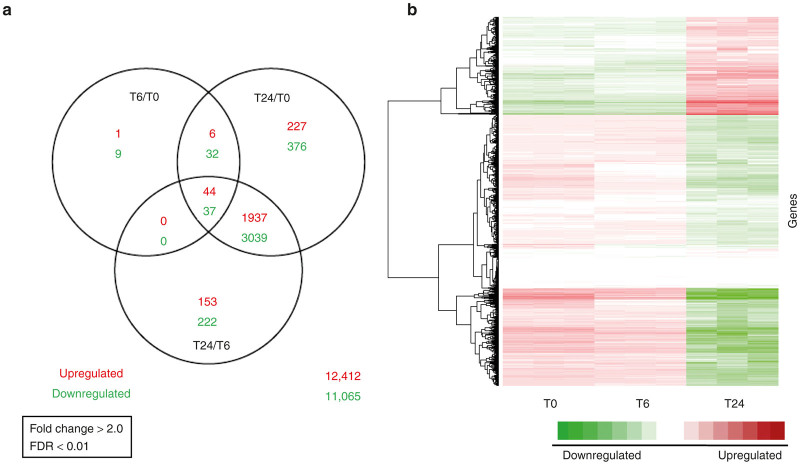
Differential gene analysis and hierarchical clustering chart/heat map. (**a**) Venn diagram demonstrating differentially up and downregulated genes using Bioconducter LIMMA package at a stricter FDR of <0.01. A total of 12,412 upregulated and 11,065 downregulated genes were identified without FDR restriction. At 6 hours postinfection, a total of 129 genes were either up- or downregulated greater than twofold (FDR <0.01), of which 124 were mapped by IPA. By 24 hours postinfection, a total of 5,698 genes were identified and 5,563 mapped by IPA. Several genes overlapped between each time point analysis, illustrated by the overlapping portions of each circle. (**b**) Heat map looking at differential changes of common genes. For this case, each gene is normalized as a mean expression of zero. Green shows samples with below mean expression and red is above mean expression. Most of the genes represented a downregulation as compared to control by 24 hours. FDR, false discovery rate.

After infecting and harvesting our samples, microarray analysis was performed. mRNA from cells were extracted, and using Affymetrix HG-U133A cDNA microarray chips, differentially expressed genes were identified using Bioconductor LIMMA statistical analysis package. A fold change of 2.0 or above was used as a cutoff, with a *P* value of 0.01. At 6 hours postinfection, a total of 129 genes were either up or downregulated greater than twofold (false discovery rate < 0.05), of which 124 were mapped by Ingenuity Pathway Analysis (IPA). By 24 hours postinfection, a total of 5,698 genes were identified and 5,563 mapped by IPA (Figure 2a; complete microarray data is available in the public Gene Expression Omnibus repository under the accession number GSE48121). The top five genes up- or downregulated at each time point are listed (Table 1). The top five significant molecular and cellular function groups (according to *P* value) with which common genes were involved entailed roles in cell morphology (11 genes, *P* = 7.06 × 10^5^), cellular development (11 genes, *P* = 2.34 × 10^4^), cellular movement (13 genes, *P* = 6.09 × 10^0^), cellular growth and proliferation (16 genes, *P* = 6.37 × 10^4^), and cell-to-cell signaling and interaction (9 genes, *P* = 6.49 × 10^4^) (Supplementary Files S1 and S2). Utilizing hierarchical clustering heat map, time-dependent gene changes are visually illustrated, with genes gradually becoming less downregulated by 6 hours to upregulated by 24 hours, and vice versa when compared to 0 hours postinfection (Figure 2b).

### Network analysis

**Figure 3:**
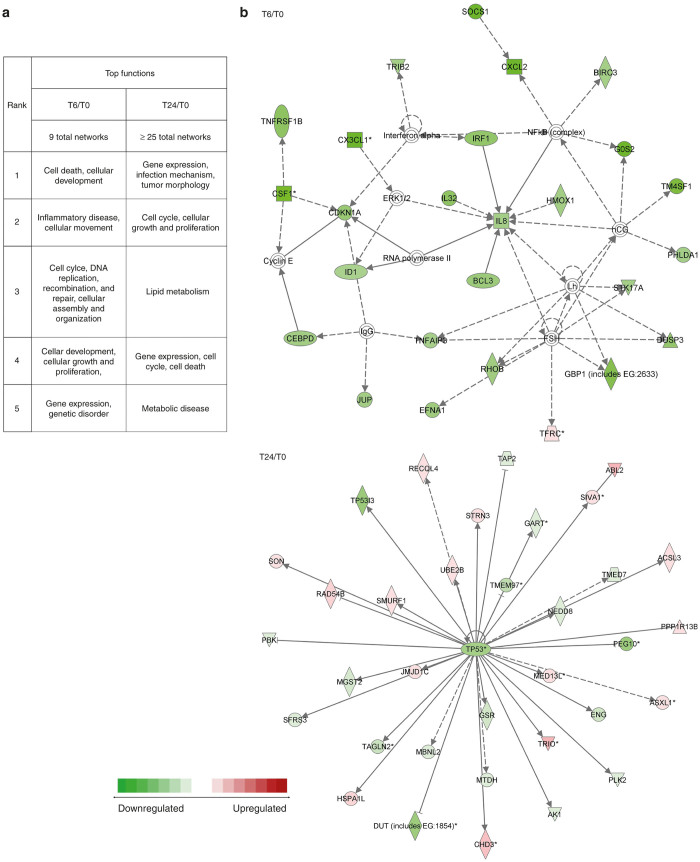
Network analysis. (**a**) Nine major networks were then identified by 6 hours, and by 24 hours, more than 25 networks were identified utilizing involved genes at each time point. (**b**) Top network at 6 hours postinfection. The top network at 6 hours postinfection included genes with functions related to Cell Death and Cellular Development and involved mostly downregulated genes such as *Il8, hmox1, bcl3, BIRC3, cxcl2, IRF1, cx3cl1, cdkna* (26 genes). (**c**) Top network at 24 hours postinfection which demonstrated mostly downregulation of gene expression. At 24 hours postinfection, the top network functions involved gene expression, infection mechanism, and tumor morphology and involved up- and downregulated genes such as *TP53, GSR, TRIO, HSPA1L, PLK2, ABL2* (35 genes).

The IPA software system enables systemic analysis of microarray and other data in a biologic context. Our up- or downregulated genes at each time point were overlaid onto a global molecular network developed from information contained in the Ingenuity Pathways Knowledge Base. Networks of these focus genes were then algorithmically generated based on their interrelationships. Nine major networks were identified by 6 hours, and by 24 hours, more than 25 networks were identified utilizing involved genes at each time point (Figure 3a, Supplementary Files S1). The top network at 6 hours postinfection included genes with functions related to Cell Death and Cellular Development and involved mostly downregulated genes such as *Il8, hmox1, bcl3, BIRC3, cxcl2, IRF1, cx3cl1, cdkna* (26 genes). At 24 hours post infection, the top network functions involved Gene Expression, Infection Mechanism, and Tumor Morphology and involved up and downregulated genes such as *TP53, GSR, TRIO, HSPA1L, PLK2, ABL2* (35 genes) (Figure 3b).

### Gene function analysis

**Figure 4:**
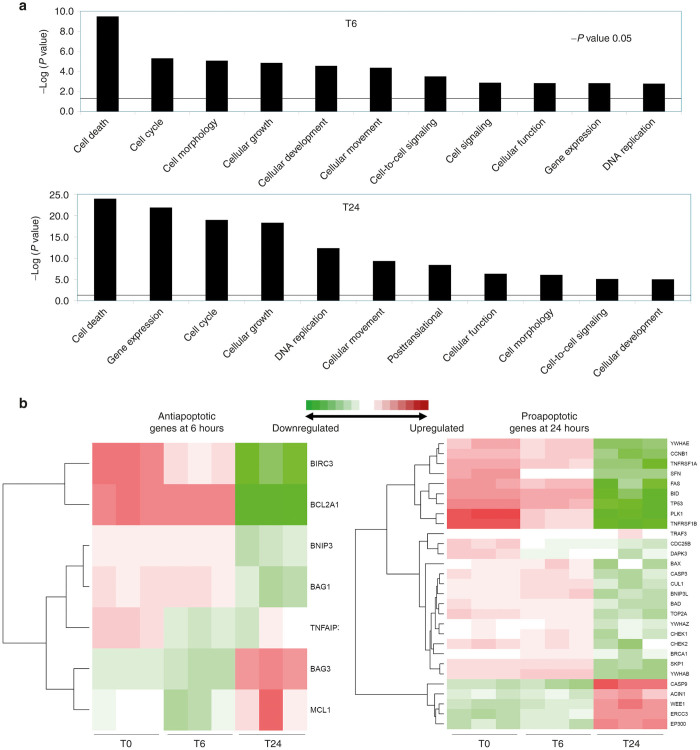
Gene function analysis. (**a**) Graph showing top cellular functions at 6 and 24 hours post infection. Bars above the line are statistically significant (*P* < 0.05). (**b**) Closer look at cell death showing important genes at 6 hours (left) mainly reflecting antiapoptotic genes, important genes by 24 hours (right) mainly reflecting proapoptotic genes. Red colored genes are overexpressed as compared to parental line; green are underexpressed.

We then investigated our overall gene list more closely and analyzed these genes in terms of some important gene and cellular functions. These graphs shows some of the functions deemed associated to our genes. All bars above the line on the graph has a *P* value less than 0.05. Statistical significance was based on the ratio of up or downregulated genes in our data set to all genes involved in the pathway. At 6 hours, important and statistically significant gene functions included cell death, cell cycle, cell morphology, growth, and development. By 24 hours after infection, cell death was still the top-rated function but also included further functions such as cell expansion and DNA repair and recombination (Figure 4a). Looking at cell death and apoptosis more closely, statistically significant genes at 6 hours involved mainly antiapoptotic pathways, with downregulation of antiapoptotic genes such as BIRC3, and TNF1IP3. By 24 hours, there is shift to modulation of genes involved in proapoptotic mechanisms, such as underexpression of BID, BAX, casp 3, and BAD creating an overall antiapoptotic state (Figure 4b, Supplementary Files S1 and S2).

### Pathway analysis

**Figure 5:**
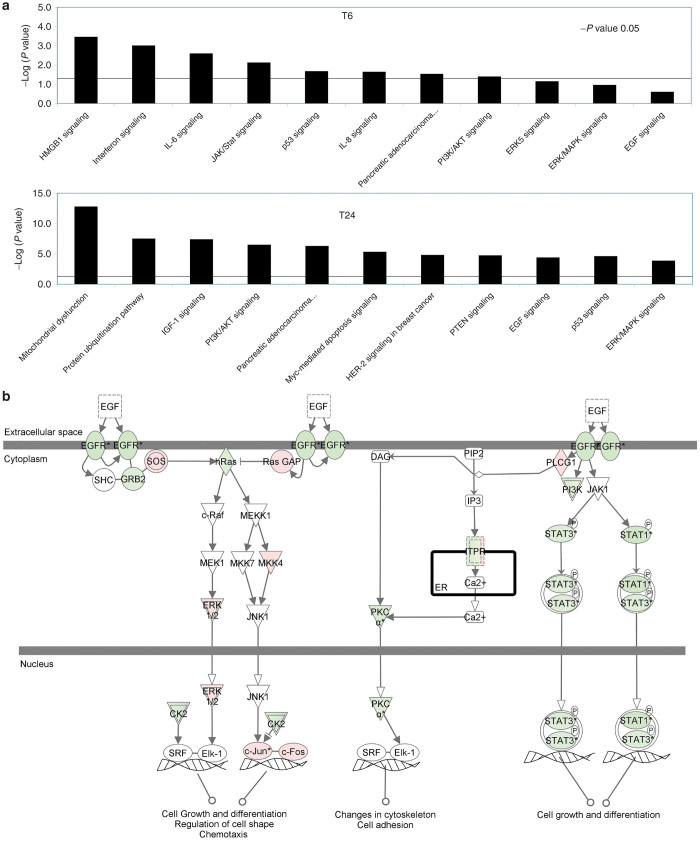
Gene pathway analysis. (**a**) Graph showing top scoring canonical pathways. (**b**) EGFR pathway. Red colored genes are overexpressed as compared to parental line; green are underexpressed. Bars above the line are statistically significant *P* < 0.05.

Canonical pathways were then identified and analyzed from the IPA libraries that were most significant to our common gene data set. The significance of the association between the data set and the canonical pathway was measured in two ways: (i) a ratio of the number of genes from the data set that map to the pathway divided by the total number of genes that map to the canonical pathway is displayed and (ii) Fischer’s exact test was used to calculate a *P* value determining the probability that the association between the genes in the dataset and the canonical pathway is explained by chance alone. IPA identified 52 genes eligible for pathways analysis. Top statistically significant canonical pathways included HMGB-1, interferon, IL-6, and JAK/STAT, signaling at 6 hours postinfection, and by 24 hours prominent pathways included PI3/AKT, epidermal growth factor receptor (EGFR), and extracellular signal-regulated kinases/mitogen-activated protein kinases (ERK/MAPK), signaling pathways (*P* < 0.05; Figure 5b). Some pathways were activated early and became more active by 24 hours, such as the EGFR pathway, an implicated pathway in pancreatic cancer resistance to conventional therapy (Figure 5b)

## Discussion

Vaccinia is a broad-spectrum virus known to infect a wide range of cell types.^5–7^ Conditionally replicating viruses have been gaining increasing attention for their ability to kill tumor cells by oncolysis and apoptosis, and hence, this attenuated strain has shown promise as a selective anticancer agent.^5^ We constructed a new virus which had three nonessential viral genes deleted and replaced with GFP, β-galactosidase, and the hNIS genes, creating GLV-1h153. hNIS is a symporter which facilitated uptake of radioiodine into GLV-1h153-infected cells, therefore enabling imaging of viral replication *in vivo* utilizing deep tissue imaging modalities such as positron emission tomography and single photon emission computed tomography.^2,8,9^ The virus also killed pancreatic cells both in cell culture and in animal models.

However, there remains a need to understand molecular mechanisms related to oncolytic viral infection and sensitivity to treatment. Therefore, this study investigated time-dependent changes in gene expression patterns associated with infection and susceptibility of pancreatic cancer cells to GLV-1h153. Identification and targeting these gene expression changes are needed to avoid side effects associated with higher doses of virus treatment, help develop new strategies to overcome resistance, and identify candidates for clinical trials.

Mechanisms of oncotherapy are still poorly understood.^10^ The proposed theory of anticancer effects appears to be mainly through oncolysis, although other cellular mechanisms such as apoptosis, necrosis, and autophagy, as well as modification to the tumor vasculature and microenvironment, may also play a role. A study by Weibel *et al*. investigated the contribution of the tumor vasculature and host immune response after therapy of breast cancer xenografts with GLV-1h68, the parent virus of GLV-1h53, and found that VACV-mediated oncolysis was the primary mechanism of tumor shrinkage in the late regression phase with neither the destruction of the tumor vasculature nor the massive VACV-mediated intratumoral inflammation being a prerequisite for tumor regression.^11^ Response of several cancer cell lines to GLV-1h68 was also investigated by Ascierto *et al*. and showed highly heterogeneous permissivity to VACV infection among the cell lines, but no clear transcriptional pattern could be identified as predictor, suggesting multifactorial basis for permissivity to viral infection.^12^ Time and MOI also appear to be important in the effectiveness of viral infection and treatment in a study by Oberg *et al*.^13^ utilizing smallpox and peripheral blood cells from human subjects. They determined that the optimal time to assess the largest amounts of gene changes was at an MOI of 0.5 PFU/cell, and at 18 hours postinfection. It has been recently shown that GFP-marker gene expression correlated with viral copy number is several cell lines, and we thus harvested infected and control pancreatic cells at a maximum of 24 hours postinfection, as determined by maximal GFP expression.^11^

It is well known that VACV infection causes significant alterations in cell function and metabolism and interferes with host cell DNA and RNA expression.^14^ Our differential gene analysis revealed a total of 12,412 up- and 11,065 downregulated genes at 6 and 24 hours postinfection with GLV-1h153 as compared to control. The top network at 6 hours postinfection included genes with functions related to cell death and cellular development and involved mostly downregulation of genes such as *IL-8, hmox1, bcl3, BIRC3, cxcl2, IRF1, cx3cl1, cdkna*. At 24 hours postinfection, the top network functions involved gene expression, infection mechanism, and tumor morphology and involved up- and downregulated genes such as *TP53*, *GSR*, *TRIO*, *HSPA1L*, *PLK2*, *ABL2*. Looking at cell death and apoptosis more closely, it was interesting that at 6 hours, the focus is on genes involved in antiapoptotic pathways, with downregulation of antiapoptotic genes such as *BIRC3*, and *TNF1IP3*. By 24 hours, there is shift to modulation of genes involved in proapoptotic mechanisms, such as underexpression of *BID, BAX, casp 3,* and *BAD* creating an overall antiapoptotic state. Antiapoptotic mechanisms early in infection may facilitate enhanced viral replication and possibly increased therapeutic efficacy, and several other studies have shown VACV’s ability to inhibit apoptosis.^15,16^ In a recent study assessing the permissivity of cell lines to GLV-1h68, *casp3* was initially downregulated, again suggesting that antiapoptosis may help facilitate viral replication.^11^

There is limited data on specific genes found to be associated with VACV infection, and none looking at pancreatic adenocarcinoma specifically.^13,17–20^ For example, Guerra *et al*. identified upregulation of genes in two clusters containing 20 immune response genes at 2, 6, and 16 hours postinfection in Human HeLa cells in response to modified vaccinia virus Ankara.^19^ Some of the key immune response genes belonging to these two clusters, including IL-1A, IL-6, IL-8, and components of signal transduction pathways, such as NFKB2, were identified in our study. Agrawal *et al*. found that vaccinia infection of dendritic cells induced the secretion of IL-6 and TNF-α, which in turn stimulated IFN-γ secretion from T cells, also upregulated in our study.^21^

We then investigated the involvement of these genes in known cell signaling pathways. Our analysis showed several statistically significant pathways including HMGB-1, IFN, IL-6, and JAK/STAT signaling at 6 hours postinfection, and by 24 hours, prominent pathways included PI3/AKT, EGFR, and ERK/MAPK, signaling pathways. Overexpression of EGFR has been implicated in pancreatic carcinoma aggressiveness and resistance to chemo- and radiation therapy.^22,23^ In a recent study by Morgan *et al*.^24^, EGFR inhibitors cetuximab and erlotinib was used in combination with gemcitabine for enhanced efficacy against pancreatic cancer. This pathway was significantly modulated by 24 hours after infection with GLV-1h153 in our study, with downregulation of the EGFR receptor, potentially mimicking effects of EGFR inhibitors.

These pathways were also involved in several studies investigating the parent virus of GLV-1h153, GLV-1h68 mostly in tumor xenografts. Worschech *et al*. demonstrated several pathways also common in our study including interferon, IL-6, JAK/STAT, PTEN, and ERK/MAPK signaling.^25^ Only purine metabolism was a common pathway with Reinboth *et al*.’s study.^26^ Like Worschech *et al*., most of our postinfection cellular gene changes reflected a downregulation and shut down of cellular metabolism.^25^ It is difficult to directly compare changes in gene expression due to RNA isolation from tissue rather than cells, adding the variable of the host immune response and gene expression changes which cannot be assessed in cell culture.

Moreover, attenuation of our virus, GLV-1h153, also likely has an important impact on results demonstrated. GLV-1h153 is a derivative of GLV-1h68, which in turn was derived from wild-type LIVP virus.^27^ Like GLV-1h153, GLV-1h68 carries three separate insertions in the F14.5L, J2R (TK), and A56R (hemagglutinin) loci of LIVP genome. Zhang *et al.* demonstrated that gene insertions in GLV-1h68 greatly reduced the replication of GLV-1h68 in normal mouse cells, whereas the replication of GLV-1h68 in tumor cells was not detrimentally affected. Furthermore, i.v. injection of GLV-1h68 into nude mice with human breast tumor xenografts showed enhanced preference for colonization of tumors when compared with wt LIVP and WR strains, leading to restricted distribution of GLV-1h68 mostly to tumors but not to other organs and therefore resulted in less toxicity and extended survival of tumor-bearing nude mice.

In addition, another paper demonstrated via experiments using wild-type single, double, and triple mutant LIVP viruses that infection and replication of LIVP mutant viruses were not very different from those of wt LIVP, suggesting that the single, double, or triple insertions within the LIVP genome did not detrimentally affect the entry and replication of the virus in tumor cells.^28^ In normal cells, however, the replication capacity of LIVP mutant viruses was greatly reduced or diminished compared with its wt strain. In addition, virus replication efficiency increased with removal of each of the expression cassettes F14.5L, J2R (TK), and A56R. The increase in virus replication efficiency was also show to be proportionate to the strength of removed VACV promoters linked to foreign genes, and replication efficiency of the new VACV strains *in vivo* paralleled their cytotoxicity in cell cultures. The authors therefore concluded that replication efficiency of oncolytic VACV in cell cultures can predict the virulence and therapeutic efficacy in nude mice, demonstrating the importance of *in vitro* experiments for further understanding of possible viral interactions *in vivo.*

However, Zhang *et al*. demonstrated via Affymetrix mouse arrays and experiments with breast tumor xenografts at 3 and 6 weeks post viral treatment with GLV-1h68 that genes denoting infiltration and activation of immune cells were strongly detected, which are expressed on activated T cells, natural killer cells, macrophages, granulocytes, and dendritic cells and associated with leukocyte activation and natural killer cytolytic function, suggesting a mouse-related immune response is part of the process leading to breast tumor regression. Preferential activation of proinflammatory transcripts, such as chemokine ligands, IL, and chemokine receptors, as well as a panel of IFN-stimulated genes was also seen. This highlights the importance of applying molecular methodologies *in vivo* in a tumor microenvironment that is as close to clinical application as possible, as it is likely that the innate and adaptive immune responses will also play a role in the way cells behave in response to infection to GLV-1h153. However, prior to conducting such experiments, it would be important to start with a more simplistic approach in cell culture, in order to be in a position to better understand more complex and intertwined gene expression changes and interactions.

Several databases investigating genes associated with poxvirus infection have been established, such as the virus pathogen database and analysis resource (ViPR),^5^ the poxvirus bioinformatics resource center,^6^ the poxvirus proteomics database,^7^ as well as literature addressing detection of and identification of different strains of orthopoxvirus.^1,29–32^ Although these databases were aimed mainly at the risk of bioterrorism or possible virus pandemic, information from these databases may be crucial to better understand virus behavior for oncotherapy.

In summary, this study reveals the ability to assess time-dependent changes of gene expression patterns in pancreatic cancer cells associated with infection and susceptibility to vaccinia viruses. Our study suggests that molecular assays may be useful to develop safer and more efficacious oncolytic viral therapies and that oncolytic viral treatments may target pathways implicated in pancreatic cancer resistance to conventional therapies.

However, we do recognize limitations of our study. Further work is needed to characterize the role of individual genes and pathways in viral therapy susceptibility and possible resistance, and to confirm these genes on a protein and translational level. Moreover, these experiments were conducted in cellular culture, and further work is necessary to establish how pancreatic cancer cells may behave in a biological model, possibly with an intact innate and adaptive immunity in place. This investigation provides a list of genes and pathways for further detailed studies and provides a framework for the observation of possible cellular events, in addition to potential biologic and molecular targets to overcome oncolytic viral resistance.

## Materials and Methods

### Virus and cell culture

African green monkey kidney fibroblast CV-1 cells and human pancreatic ductal carcinoma PANC-1 cells were purchased from American Type Culture Collection (Manassas, VA) and were grown in Dulbecco’s modified Eagle’s medium (DMEM) supplemented with 1% antibiotic–antimycotic solution (Mediatech, Herndon, VA) and 10% fetal bovine serum (Mediatech) at 37 °C under 5% CO_2_. GLV-1h68 was derived from VACV LIVP, as described previously.^27^ GLV-1h153 was derived from GLV-1h68, as also previously described.^27^

### Flow cytometry

Cells were seeded on six-well plates at 5 × 10^5^ cells per well. Wells were then infected at multiplicities of infection (MOIs) of 0, 0.01, 1.0, and 5, and cells then harvested at 6 and 24 hours postinfection by trypsinizing and washing with phosphate-buffered saline. GFP expression was analyzed via a Becton-Dickinson FACScan Plus cytometer (Becton-Dickinson, San Jose, CA). Analysis was performed using CellQuest software (Becton-Dickinson). Three samples were performed at each time point and averaged.

### Preparation of RNA for microarray

Total mRNA preparation was performed in six-well culture plates. Cells were plated at 5 × 10^5^ cells per well and infected with GLV-1h153 at an MOI of 5.0. Zero, 6, and 24 hours postinfection, three samples of at each time point were harvested and lysis performed directly using RNeasy mini kit protocol (Qiagen, Valencia, CA). The mRNA samples were measured by spectrophotometer for proof of purity and hybridized to HG-U133A cDNA microarray chips (AffymetrixInc, Santa Clara, CA) by the genomic core laboratory at Memorial Sloan-Kettering Cancer Center.

### Microarray analysis

The HG-U133A cDNA microarray chip images were scanned and processed to CEL files using the standard GCOS analysis suite (AffymetrixInc). CEL files were then normalized and processed to signal intensities using the gcRMA algorithm from the Bioconductor library for the R statistical programming system. All subsequent analysis was done on the log (base 2) transformed data. To find differentially expressed genes a moderated *t*-test was used as implemented in the Bioconductor LIMMA package. To control for multiple testing the false discovery rate method was used with a cutoff of 0.05.

## Figures and Tables

**Table 1:** Genes up and downregulated in GLV-1h153-infect PANC-1 cells

*Probe ID*	*Name*	*Symbol*	*Fold change*	*Average FDR*
T6/T0 Upregulated				
211123_at	Solute carrier family 5 (sodium iodide symporter), member 5	SLC5A5	1,893.82	4.55496 × 10^8^
207046_at	Histone cluster 2, H4a	HIST2H4A	161.32	5.38612 × 10^5^
216796_s_at	AK026847	AK026847	113.89	5.08978 × 10^6^
206951_at	Histone cluster 1, H4e	HIST1H4E	91.54	9.22164 × 10^8^
214516_at	Histone cluster 1, H4b	HIST1H4B	73.95	1.03942 × 10^7^
T6/T0 downregulated				
201169_s_at	Basic helix-loop-helix domain containing, class B, 2	BHLHB2	−4.67	0.000984296
823_at	chemokine (C-X3-C motif) ligand 1	CX3CL1	−4.37	0.000173528
203687_at	chemokine (C-X3-C motif) ligand 1	CX3CL1	−4.33	0.001296406
213524_s_at	G0/G1switch 2	G0S2	−4.32	0.002112328
210001_s_at	Suppressor of cytokine signaling 1	SOCS1	−4.3	0.001525627
T24/T0 upregulated				
211123_at	Solute carrier family 5 (sodium iodide symporter), member 5	SLC5A5	4,935.22	1.59464 × 10^9^
216796_s_at	AK026847	AK026847	1,314.03	2.21339 × 10^8^
117_at	Heat shock 70 kDa protein 6 (HSP70B’)	HSPA6	1,106.5	2.24183 × 10^9^
213418_at	Heat shock 70 kDa protein 6 (HSP70B’)	HSPA6	355.86	2.2579 × 10^8^
207046_at	Histone cluster 2, H4a	HIST2H4A	315.33	5.72591 × 10^7^
T24/T0 downregulated				
211506_s_at	Interleukin 8	IL8	−140.35	3.6625 × 10^6^
202638_s_at	Intercellular adhesion molecule 1 (CD54), human rhinovirus receptor	ICAM1	−116.46	4.02096 × 10^6^
202037_s_at	Secreted frizzled-related protein 1	SFRP1	−101.92	3.50598 × 10^11^
205476_at	Chemokine (C-C motif) ligand 20	CCL20	−86.1	1.08182 × 10^6^
201980_s_at	Ras suppressor protein 1	RSU1	−81.93	6.84573 × 10^9^

FDR, false discovery rate.

## Notes

N.C., Q.Z., and A.Z. are affiliated with Genelux Corporation. No competing financial interests exist for D.H., C-H.C., N.S., S.C., A. M. and Y.F.

The last two authors are co-corresponding authors.
